# Optimization of irrigation and fertilization of apples under magnetoelectric water irrigation in extremely arid areas

**DOI:** 10.3389/fpls.2024.1356338

**Published:** 2024-03-20

**Authors:** Xiaoxian Duan, Quanjiu Wang, Weiyi Mu, Xuesong Wei

**Affiliations:** State Key Laboratory of Eco-hydraulics in Northwest Arid Region, Xi’an University of Technology, Xi’an, China

**Keywords:** apple, magnetoelectric water, quality, photosynthetic characteristics, yield, arid areas

## Abstract

Apple (*Malus pumila Mill.*) is one of the important economic crops in the arid areas of Xinjiang, China. For a long time, there has been a problem of high consumption but low yield in water and fertilizer management, prevent improvements in apple quality and yield. In this study, 5-year-old ‘Royal Gala’ apple trees in extremely arid areas of Xinjiang were used as experimental materials to carry out field experiments. considering 5 irrigation levels (W1, 30 mm; W2, 425 mm; W3, 550 mm; W4, 675 mm; W5, 800 mm) and 5 fertilization levels (F1, 280 kg·ha^-1^; F2, 360 kg·ha^-1^; F3, 440 kg·ha^-1^; F4, 520 kg·ha^-1^; F5, 600 kg·ha^-1^) under magnetoelectric water irrigation conditions. The results demonstrated that magnetoelectric water combined with the application of 675 mm irrigation amount and 520 kg·ha^-1^ fertilization amount was the most effective combination. These results occurred by increasing net photosynthetic rate of apple leaves, improved the quality of apples, increased apple yield, and promoted the improvement of water and fertilizer use efficiency. Additionally, the quadratic regression model was used to fit the response process of yield, IWUE and PFP to irrigation amount and fertilization amount, and the accuracy was greater than 0.8, indicating good fitting effects. The synergistic effect of water and fertilizer has a positive effect on optimizing apple water and fertilizer management. Principal component analysis showed that the magnetoelectric treatment combined water and fertilizer mainly affected apple yield, water and fertilizer use efficiency and vitamin C content related to quality. This study provides valuable guidance for improving water and fertilizer productivity, crop yield and quality in extreme arid areas of Xinjiang by using Magnetoelectric water irrigation.

## 1 Introduction

Apple has the characteristics of strong adaptability and high nutritional value. It is one of the main economic crops all over the world (Hou et al., 2021). China is the world ‘s largest apple producer and consumer. According to statistics, China ‘s apple production will reach 44,066,100 t in 2020, accounting for 47% of global production (Peng et al., 2023b). Due to its unique climatic conditions, southern Xinjiang has gradually become the main apple planting base in China. However, the development of the apple industry in this region is severely constrained by natural conditions such as water scarcity and soil salinization (Wang and Liu, 2022). Therefore, optimizing the management mode of water and fertilizer, enhancing the efficiency of water and fertilizer utilization have become the key issues facing the development of apple planting industry in southern Xinjiang, China (Zhang et al., 2015). Recently, the development and application of agricultural water-saving and fertilizer-saving technologies have led to a certain improvement in the efficiency of water and fertilizer utilization in agriculture. This is primarily achieved by regulating the use of a single component, either water or fertilizer. We believe that attention should be paid to the physicochemical properties of water itself and the synergistic effects between water and fertilizer, these two key points. Initiating in-depth research and developing a novel water and fertilizer management technology based on these aspects will be an important subject (Friedman and Naftaliev, 2012; Esmaeilnezhad et al., 2017).

Activated irrigation water is to treat irrigation water by means of magnetization, electron removal and oxygenation to improve the surface tension, dissolved oxygen and other physical and chemical properties of irrigation water, so as to improve the activity of irrigation water and enhance the physiological effect of irrigation water. Magnetized water occurs when a body of water passes through a fixed magnetic field environment at a certain flow rate perpendicular to the magnetic lines of force (Xiaofeng and Bo, 2007; Wang et al., 2016). Studies have shown that after the water is magnetized, the average distance between water molecules increases, some hydrogen bonds become weaker or even broken, large associative water molecular clusters become smaller, and the number of free monomer water molecules and dimer water molecules in the water increases, resulting in increased osmotic pressure and solubility, and decreased viscosity coefficient and surface tension (Xiao-Feng and Bo, 2008). Under the condition of static magnetic field, the evaporation of water increases, and the increase in mass evaporation depends on the magnetization (Ben Amor et al., 2022). In the field of agriculture, magnetized water treatment technology has also been widely studied and applied. Research shows that magnetized saline irrigation water, even at high salinity, increases barley growth parameters as well as photosynthetic pigments, resulting in an increase in grain yield compared to irrigation with non-magnetized saline water (Hozayn et al., 2021). Irrigation of wheat with magnetized water reduced the harmful effects of salinity in all studied factors, Moreover, the Bayesian inference disclosed that whatever the salinity rate is, the positive impact of the magnetic field on growth and yield factors is obvious. the use of magnetized water is highly recommended for land irrigation (Ben Hassen et al., 2020). De-electronized water refers to the line connected to the earth on the metal pipe. When the water flows through the metal pipe, the electrons in the water body are enriched on the pipe wall, and the physical and chemical properties of the irrigation water are changed by introducing the electrons into the ground (Duan et al., 2022). Research showed that de-electronized water with high salinity, the negative ions in the water body were effectively eliminated, and the proportion of positive ions was greatly increased, thus affecting the characteristics of soil water and salt transport (Wang et al., 2016, 2018). Compare to conventional irrigation, de-electronized water irrigation increased the yield per plant and water use efficiency (WUE) of winter wheat (*Triticum aestivum L.*) by 17.8% and 15.1% (Wang et al., 2020). De-electronized water was added during aerobic composting was shorten the period of compost, augment the relative abundance of advantage genus, and then enhance the stability of compost products (Duan et al., 2022). Both magnetization technology and electron removal technology are helpful to agricultural production. The ways and means of their effects on irrigation water are different, but the effects are similar. The study found that compared with single application, the coupling application of magnetization and de-electronic technology has a better effect on improving the physical and chemical properties of water bodies, and can maintain the improved physical and chemical properties of irrigation water for a long time (Li et al., 2021). At present, the research on magnetization and de-electronic coupling treatment technology in agricultural irrigation is still in its infancy, and there are few related research reports. Therefore, it is necessary to carry out related research (Wang et al., 2019a).

There is a strong interaction between water and nutrients, which may have positive or negative effects on crops according to the types of crops, growth stages and planting environments (Shi et al., 2023). Therefore, the use of water and fertilizer coupling to optimize crop water and fertilizer management plays an important role in improving water and fertilizer use efficiency. Studies have shown that increasing the supply of fertilizer is beneficial to alleviate the metabolic disorder of plants under water shortage and improve the resistance of plants to drought (Dou et al., 2022). Under the condition of normal water supply, reasonable fertilization increased the ratio of transpiration water loss to evapotranspiration water loss, reduced evaporation water loss, and improved water use efficiency (Yang et al., 2023). Nitrogen, phosphorus and potassium contained in agricultural fertilizers are necessary substances for crop growth (Wang et al., 2023). Increasing the supply of fertilizer can increase the chlorophyll content of leaves, protect photosynthetic organs from the influence of drought and water shortage, so that photosynthetic organs can give full play to their role, thus increasing photosynthesis (Duan and Chang, 2017). Appropriate water management can increase the effectiveness of nutrients. Studies have shown that sufficient soil water content can promote the nitrification of ammonium nitrogen, and when the water content is too high or too low, the process will be inhibited (Wang et al., 2023). Soil water content can affect the absorption of mineral nutrients by plant roots, and further affect the growth of shoots and the formation of yield (Zhang et al., 2017). Through sufficient irrigation, the difference in nitrate nitrogen concentration at different positions of the soil is reduced, which is a typical example, indicating that nutrients can be transferred to plant roots through water movement. Sufficient soil water content can significantly transfer most of the nutrient ions to the aboveground part and increase the nutrient content of the plant. Studies have shown that drip fertigation can maintain high root activity and improve the ability of fruit trees to absorb water and nutrients, thereby promoting the yield of fruit trees (Peng et al., 2023a). Under the condition of drip irrigation and fertilization, excessive irrigation and fertilization will not increase the yield, but will lead to the accelerated vegetative growth of apple trees and affect the improvement of water use efficiency (Zhu et al., 2023). In previous studies, domestic and foreign scholars have inconsistent conclusions on the regulation of apple fruit quality by irrigation and fertilization. It was found that when appropriate nitrogen fertilizer was applied, reducing irrigation would reduce vitamin C and soluble solids, but could increase total soluble sugar concentration and titratable acidity (Sun et al., 2022). In addition, studies have shown that water deficit contributes to the improvement of soluble sugar concentration and soluble solids content (Zha et al., 2023). The response of apple quality under different water and fertilizer treatments needs further study. To promote the absorption of water and nutrients to give full play to its role, to achieve maximum yield and optimal quality, and to reduce the ecological environment pressure caused by excessive fertilization and irrigation, we should fully understand and utilize their positive interaction, not only pay attention to the input of water and nutrients, but also pay attention to their reasonable combination. Increasing the amount of irrigation should consider nutrient supply capacity, and increasing the supply of nutrients should consider water absorption capacity, so that the limited nutrients and water resources have the best effect (Wang et al., 2021).

In summary, we hypothesize that magnetoelectric water treatment and water and fertilizer coupling may be more conducive to crop growth and development in arid areas, improve crop yield and water and fertilizer use efficiency. This is of great significance to alleviate the impact of water shortage and land salinization on agricultural development in arid areas. Therefore, we have carried out relevant experimental studies to verify our previous conjectures.

## 2 Materials and methods

### 2.1 Experimental site

Field studies were conducted during 2021 at the apple garden of Alar City (40°39′14″ N, 81°16′21″ E), Xinjiang Province, Northwest China, which belongs to a typical inland extremely arid climate zone.

**Figure 1:**
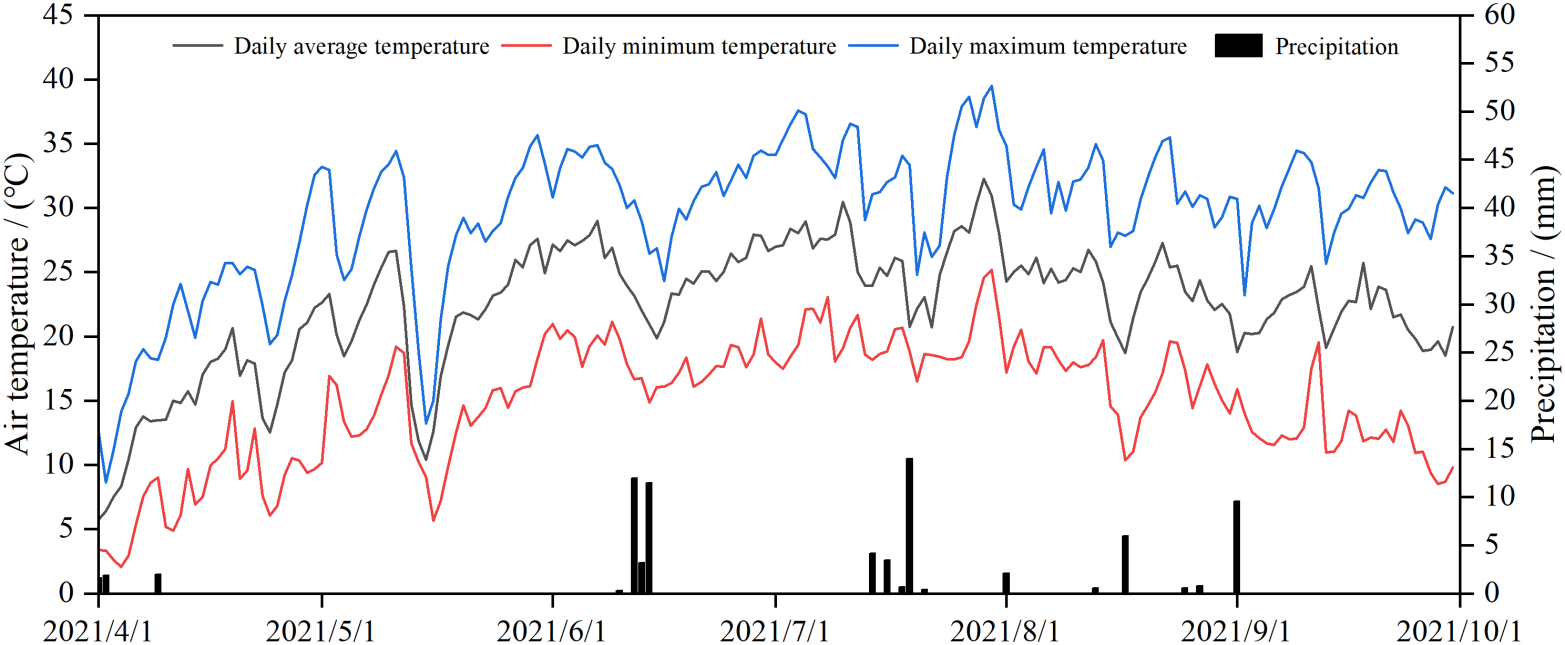
Daily air temperature and precipitation during the apple growing seasons in 2021.

We collected daily precipitation and average air temperature from an automatic meteorological station located in the field (**Figure 1**). The daily average air temperature during the apple growth period (April-September) was 22.38 °C, with a total precipitation of 128 mm, for 2021. The annual evaporation is about 2100mm, the annual sunshine hours amount to about 2900, the frost-free period is more than 200 days, and the groundwater depth is more than 3 m.

The soils were classified as sandy soil with an average particle size distribution of approximately 96.39% sand, 3.19% silt and 0.42% clay (**Table 1**). We measured the relevant soil physical and chemical properties. The average soil bulk density of 1.52 g·cm^-3^. Available phosphorus and available boron contents of 3.20 mg·kg^-1^ and 0.60 mg·kg^-1^, rapid potassium content of 33 mg·kg^-1^, alkaline nitrogen and total nitrogen contents of 10 mg·kg^-1^ and 176 mg·kg^-1^, ammonium nitrogen and nitrate nitrogen contents of 2.01 mg·kg^-1^ and 1.00mg/kg, respectively, PH of 8.71, and EC value of 154.60 μs·cm^-1^.

**Table 1:** Physical properties of 0-120 soil layers in the experimental area.

Soil depth (cm)	Bulk density (g·cm^-3^)	Clay (<0.002mm)/%	Silt (0.002~0.02mm)/%	Sand (0.02~0.2mm)/%	Soil texture
0~20	1.49	0.47	3.55	95.99	Sandy soil
20~40	1.56	1.81	8.09	90.35	Sandy soil
40~60	1.54	0	1.31	98.69	Sandy soil
60~80	1.53	0	1.39	98.61	Sandy soil
80~100	1.48	0	0.39	99.61	Sandy soil
100~120	1.51	0.49	4.42	95.09	Sandy soil

### 2.2 Experimental materials

The experiment was conducted from April to September 2021. The apple tree variety used in the experiment was “Royal Gala”, and the rootstock was M195. The tree was planted in 2015 with a planting density of 2850 plants·ha^-1^, and the height was 3~3.5 m, the plant spacing 1 m, and the row spacing 3.5m. The phenological period of the apple tree was divided into four parts: anthesis fruit setting stage (AFS) was from 20 April to 1 May, the young fruit development stage (YFS) from 2 May to 1 June, the fruit expansion stage (FES) from 2 June to 1 August, and the fruit ripeness stage (FRS) from 2 August to 20 August.

**Figure 2:**
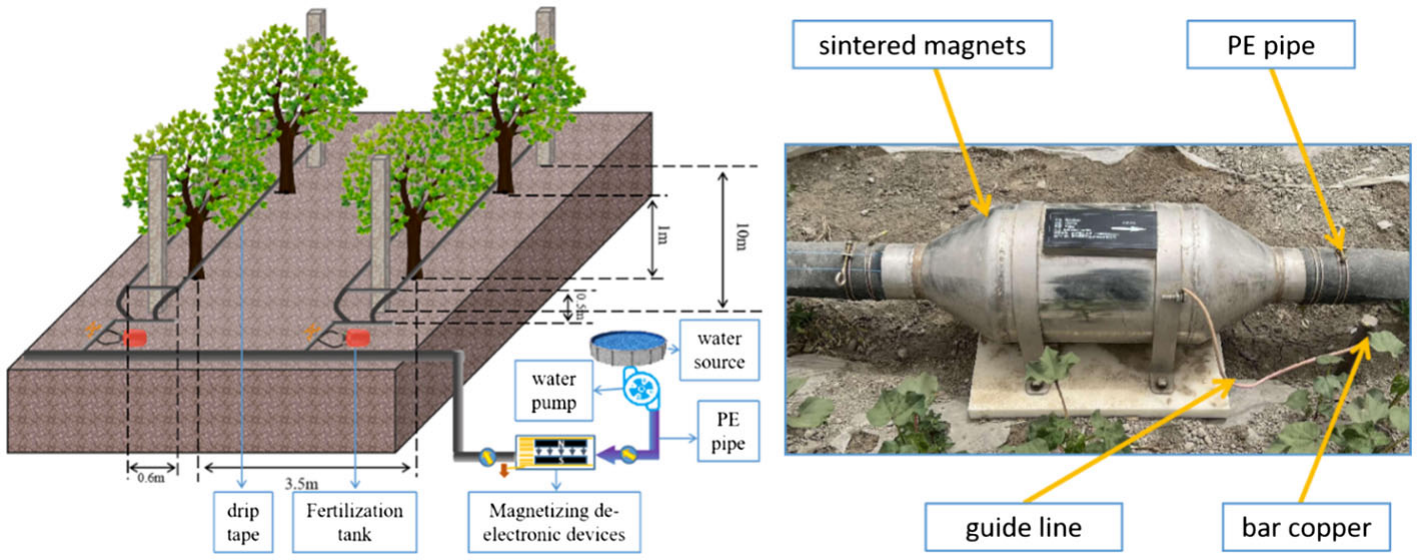
Layout of test area and magnetization and de-electronic processing devices.

We arranged a drip irrigation belt on both sides of the tree, and the distance between the two drip irrigation belts was 60 cm. The distance between the drip irrigation belt and the ground is 50 cm. The drop head flow was 4 L·h^-1^. The tanks were pressure differential fertilization tanks, which were individually configured for each test plot. The layout of the test area is shown in (**Figure 2**).

Irrigation water is fresh water after magnetization treatment and de-electronic treatment, namely of the magnetoelectric water. According to the research of Wang et al., we determined that the magnetic field strength is 3000 GS. The magnetization treatment device is composed of CHQ external magnetizer (Baotou, China). The magnetizer is made of sintered Rufe-B, and the magnetic field intensity was calibrated using a 5180 gauss meter (F.W.BELL, USA). When the fresh water passes through the permanent magnet at a fixed flow rate (0.5m·s^-1^), the magnetic induction line is cut vertically several times to ensure that the effective magnetization times are more than 10 times. After magnetization treatment, it flows into the de-electronic device. The device exports the electrons in the magnetized water through an externally connected grounding resistance to complete the preparation of experimental irrigation water (**Figure 2**).

### 2.3 Experimental design

In this experiment, before formulating the irrigation and fertilization systems, we consulted a large amount of literature and communicated with local agricultural managers to ensure the reliability of the irrigation and fertilization systems. Therefore, we set five irrigation quotas (300 mm, 425 mm, 550 mm, 675 mm, 800 mm), which were designated W1, W2, W3, W4, W5 respectively. Five fertilization quotas (280 kg·ha^-1^、360 kg·ha^-1^、440 kg·ha^-1^、520 kg·ha^-1^、600 kg·ha^-1^) were determined, which were designated F1, F2, F3, F4, F5 respectively. When applying fertilizer, the ratio of NPK fertilizer is 1: 1: 2, nitrogen is supplied through urea (≥ 46%), potassium is supplied through potassium sulfate (K_2_SO_4_ ≥ 52%) and potassium dihydrogen phosphate (K_2_O ≥ 33.8%), phosphate is supplied through potassium dihydrogen phosphate (P_2_O_5_≥51%). A completely randomized block experimental design was used with 25 treatments. There are 10 trees in each experimental plots, with an area of 35 m^2^, and the plots were spaced by pollination trees. The agronomic management including weeding, insecticide spraying, pruning flower and fruit thinning were conducted according to the local standardized orchard. Irrigation and fertilization systems are shown in **Table 2**.

**Table 2:** Irrigation quota and fertilization quota at different growth stages.

Treatments	FFS	YFS	FES	FMS	Total
W1 (mm)	15.00	75.00	180.00	60.00	300.00
W2 (mm)	21.25	106.25	255.00	85.00	425.00
W3 (mm)	27.50	137.50	330.00	110.00	550.00
W4 (mm)	33.75	168.75	405.00	135.00	675.00
W5 (mm)	40.00	200.00	480.00	160.00	800.00
F1 (kg·ha^-1^)	23.33	70.00	140.00	46.67	280.00
F2 (kg·ha^-1^)	30.00	90.00	180.00	60.00	360.00
F3 (kg·ha^-1^)	36.67	110.00	220.00	73.33	440.00
F4 (kg·ha^-1^)	43.33	130.00	260.00	86.67	520.00
F5 (kg·ha^-1^)	50.00	150.00	300.00	100.00	600.00

‘ W1 ‘, ‘ W2 ‘, ‘ W3 ‘, ‘ W4 ‘ and ‘ W5 ‘ indicate the irrigation amount of 300 mm, 425 mm, 550 mm, 675 mm and 800 mm, respectively. ‘ F1 ‘, ‘ F2 ‘, ‘ F3 ‘, ‘ F4 ‘ and ‘ F5 ‘ indicate the fertilization amount of 280 kg·ha^-1^, 360 kg·ha^-1^, 440 kg·ha^-1^, 520 kg·ha^-1^ and 600 kg·ha^-1^, respectively. FFS indicates the flowering to fruit-setting stage, YFS indicates the young fruit stage, FES indicates the fruit expanding stage, FMS indicates the fruit maturation stage.

### 2.4 Measurements and calculations

#### 2.4.1 Photosynthetic characteristics

Li-6400 portable photosynthesis systems (LI-COR, USA) were used to determine the photosynthetic parameters on cloudless day during the fruit expansion stage (FES, July 23, 2020). Three healthy leaves on new apple branches were used to determine the photosynthesis indicators at a vertical height of 1.5–1.8 m on the sunny side of each tree. The measurement time is from 9 a.m. to 11 a.m. and from 4 p.m. to 6 p.m. The photosynthetic indicators investigated in this study were the intercellular CO_2_ concentratin (Ci, µmol·m^-2^·s^-1^), stomatal conductance (Gs, mol·m^-2^·s^-1^), transpiration rate (Tr, mmol·m^-2^·s^-1^), net photosynthetic rate (Pn, µmol·m^-2^·s^-1^) of the apple leaves. Carboxylation efficiency (CE, µmol·mmol^-1^) and leaf instantaneous water use efficiency (LWUE, µmol·mmol^-1^) were calculated by Equations 1, 2, calculated as follows (Luo et al., 2023):


(1) $$C\;E=\frac{P\;n}{C\;i}$$

where Pn is the net photosynthesis rate (μmol·m^−2^·s^−1^) and Ci, that of intercellular CO_2_ concentratin (µmol·m^−2^·s^−1^).


(2) $$L\;W\;U\;E=\frac{P\;n}{T\;r}$$

where Pn is the net photosynthesis rate (μmol·m^−2^·s^−1^) and Tr, that of transpiration rate (mmol·m^−2^·s^−1^).

#### 2.4.2 Fruit quality parameters

After the apple tree entered the fruit ripeness stage, the canopy of the fruit tree was divided into three layers: upper, middle and lower. Three apples were selected in the east, south, west and north directions of each layer, and their appearance quality and chemical quality were measured.

Appearance quality parameters. Fruit weight (SW, g) was determined by electronic scales. Fruit length (H, mm) and diameter (R, mm) were determined by a vernier caliper. The fruit shape index (SI) was calculated by the ratio between fruit length and diameter.

Intrinsic quality parameters. Total soluble solids (SS) were determined by a handheld saccharimeter (AK00A). Firmness (P, kg·cm^-2^) was determined by a handheld sclerometer (GY-4). Titrated acidity (TA) was titrated with 0.1 mol/L NaOH solution. Vitamin C (VC) content was analyzed using the molybdenum blue colorimetric method (Shi et al., 2023).

#### 2.4.3 Yield

In early August of 2021, three representative apple trees were selected for each treatment, and the fruit was picked and the yield per plant was counted. According to the area of the experimental plot and the number of cultivated plants, the yield per hectare of each treatment was converted.

#### 2.4.4 Irrigation water use efficiency

Irrigation water use efficiency (IWUE) is the ratio of the yield to the amount of irrigation per unit area of apple tree and was calculated as Equation 3 (Du et al., 2017):


(3) $$I\;W\;U\;E=\frac{Y}{W}$$

where Y is the yield per unit area (kg·ha^-1^) of apple tree and W, that of irrigation amount (mm).

#### 2.4.5 Partial factor productivity

The partial factor productivity (PFP) is the ratio of apple yield per unit area to fertilizer application per unit area, calculated as Equation 4 (Dai et al., 2021):


(4) $$PFP=\frac{Y}{F}$$

where Y is the yield per unit area (kg·ha^-1^) of apple tree and F is the total amount of fertilizer applied (kg·ha^-1^).

### 2.5 Statistical analysis

Microsoft Excel 2010 was used to sort out the data. SPSS 22.0 was used to analyze the variance and significance of the data. Origin2020 was used for drawing and correlation analysis of each index. The principal component analysis, correlation analysis and regression model were carried out by matlab programming software.

## 3 Results

### 3.1 Photosynthetic characteristics

**Figure 3:**
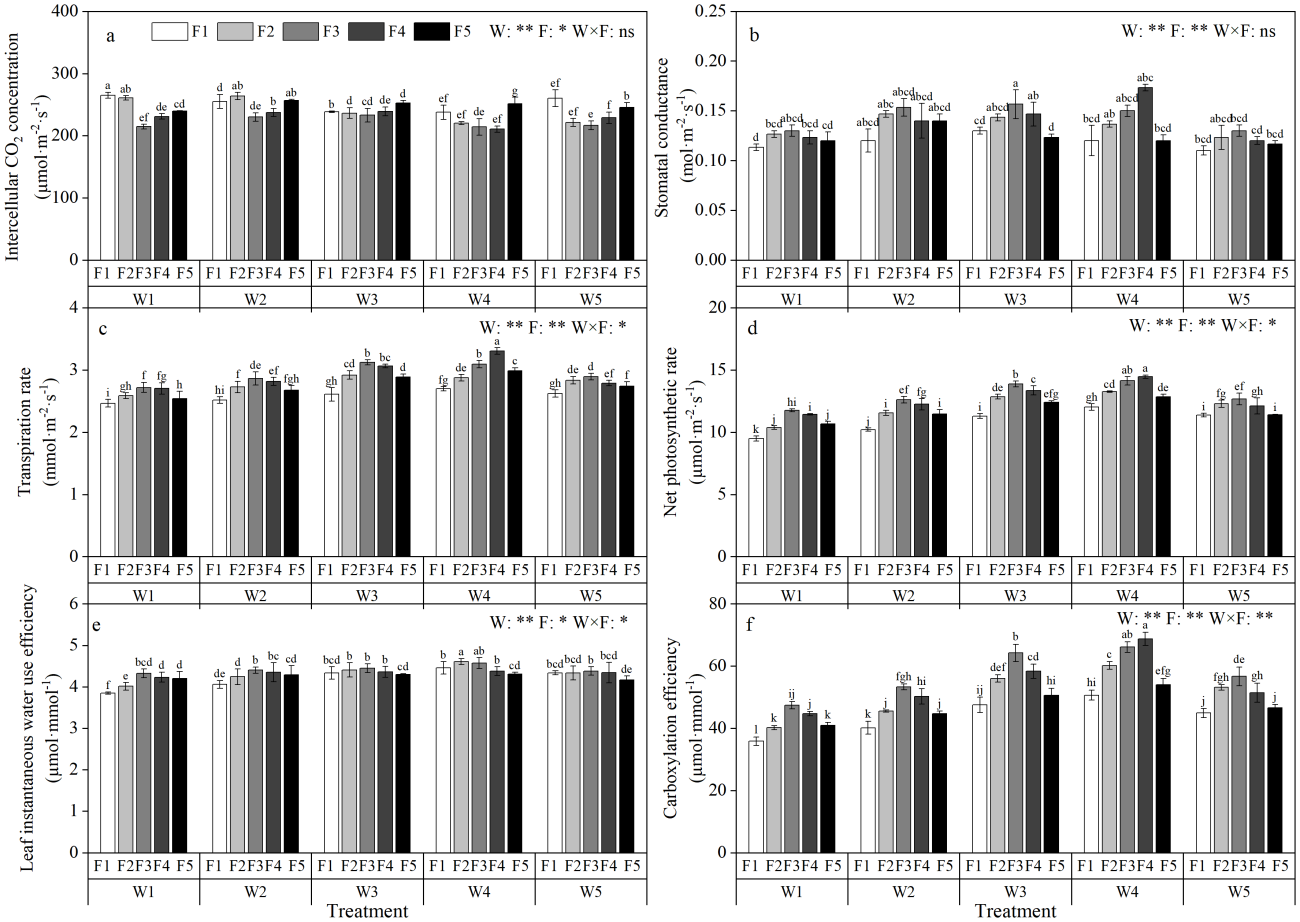
Effects of different irrigation amount and fertilization amount on photosynthetic characteristics of apple leaves under magnetic-electric water irrigation. Intercellular CO_2_ concentratin **(A)**, Stomatal conductance **(B)**, Transpiration rate **(C)**, Carboxylation efficiency **(D)**, Leaf instantaneous water use efficiency **(E)**, Net photosynthetic rate **(F)**;’ W ‘ and ‘ F ‘ indicate the amount of irrigation and fertilization respectively. ‘ W1 ‘, ‘ W2 ‘, ‘ W3 ‘, ‘ W4 ‘ and ‘ W5 ‘ indicate the irrigation amount of 300 mm, 425 mm, 550 mm, 675 mm and 800 mm, respectively. ‘ F1 ‘, ‘ F2 ‘, ‘ F3 ‘, ‘ F4 ‘ and ‘ F5 ‘ indicate the fertilization amount of 280 kg·ha^-1^, 360 kg·ha^-1^, 440 kg·ha^-1^, 520 kg·ha^-1^ and 600 kg·ha^-1^, respectively. Different letters in the same column indicate the significant difference between treatments at P = 0.05 level. * and ** indicate significant difference at P = 0.05 and P = 0.01 levels, respectively. ns indicates non-significant difference at P = 0.05 level.

The strength of photosynthesis directly reflects the growth status of crops. It can be seen from **Figure 3** that the Ci decreased first and then increased with the increase of irrigation amount and fertilization amount, and reached the minimum values of 224.13 µmol·m^−2^·s^−1^ and 227.73 µmol·m^−2^·s^−1^ under W4 irrigation amount and F3 fertilization amount, respectively. Under the interaction of water and fertilizer, W4F4 treatment reached the minimum value of 210.67 µmol·m^−2^·s^−1^, which decreased by 6.39% and 8.10% respectively compared with the minimum value under the single factor of water and fertilizer, reflecting that under the condition of insufficient water and nutrient supply, the metabolic activity inside the leaves was weakened, and CO_2_ could not be effectively transported and fixed. With the increase of irrigation and fertilization, Pn, Tr, Gs, CE and LWUE increased first and then decreased. Among them, the Pn reached the maximum values of 13.35 µmol·m^−2^·s^−1^ and 13.01 µmol·m^−2^·s^−1^ under W4 irrigation and F3 fertilization, respectively. Under the interaction of water and fertilizer, W4F4 treatment reached the maximum value of 14.67 µmol·m^−2^·s^−1^, which increased by 8.60% and 11.22% respectively compared with the maximum net photosynthetic rate under the single factor of water and fertilizer. This directly reflects that under the condition of suitable water and fertilizer, the photosynthesis of apple leaves is enhanced and the plant growth state is better. The effects of irrigation amount on Pn, Tr, Gs, Ci, CE and LWUE reached extremely significant levels (P<0.01). The effect of fertilization on Pn, Tr, Gs and CE reached a very significant level (P<0.01), and the effect on Ci and LWUE reached a significant level (P<0.05). In terms of synergistic effect, the interaction between irrigation amount and fertilization amount had no significant effect on Ci and Gs, but had significant effect on Pn, Tr and LWUE (P<0.05), and had extremely significant effect on CE (P<0.01) (**Figure 3**).

### 3.2 Fruit quality parameters

**Figure 4:**
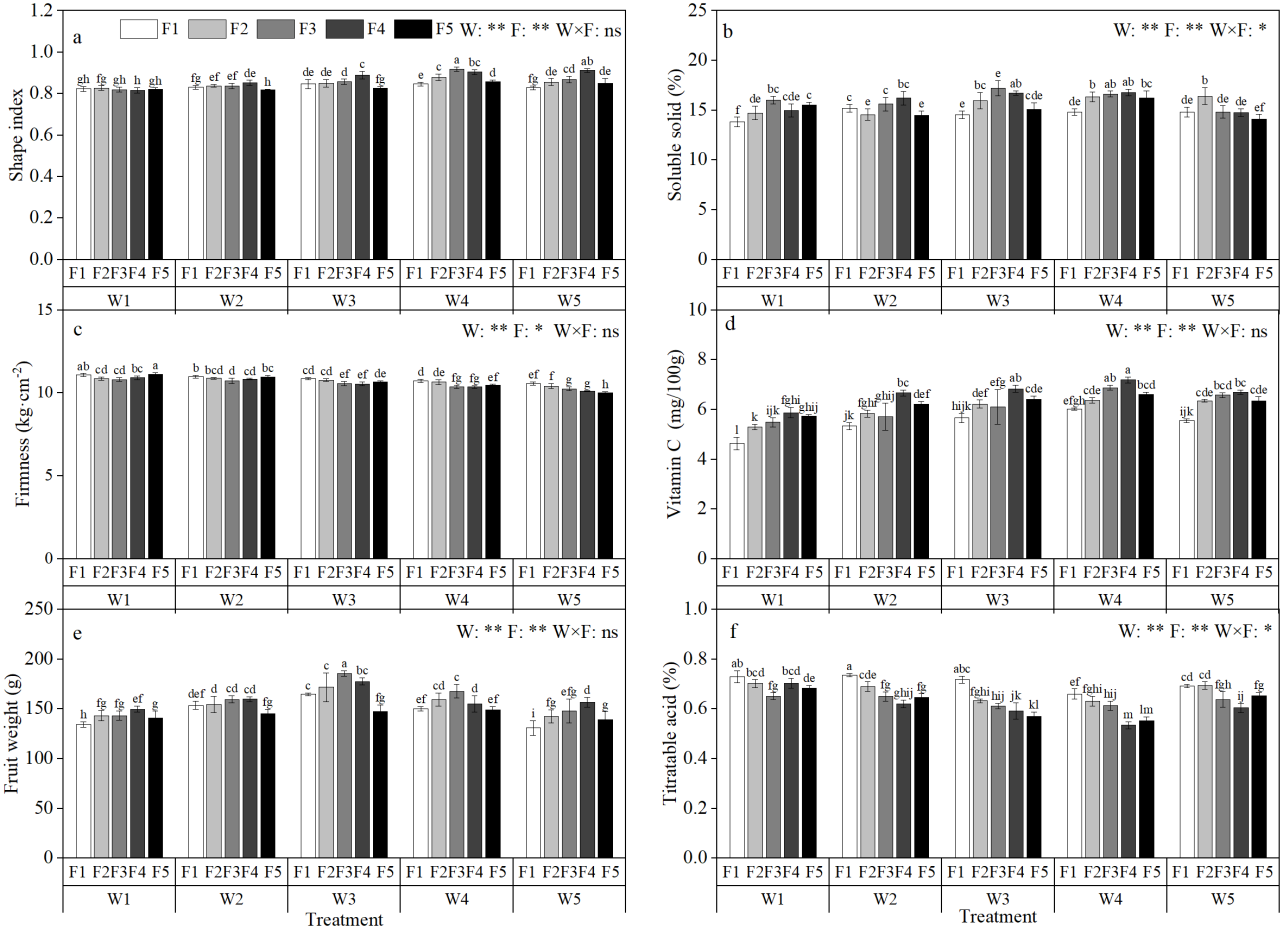
Effects of different irrigation amount and fertilization amount on the quality of apples under magnetic-electric irrigation. Shap index **(A)**, Soluble solid **(B)**, Firmness **(C)**, Vitamin C **(D)**, Fruit weight **(E)**, Titratable acid **(F)**;’ W ‘ and ‘ F ‘ indicate the amount of irrigation and fertilization respectively. ‘ W1 ‘, ‘ W2 ‘, ‘ W3 ‘, ‘ W4 ‘ and ‘ W5 ‘ indicate the irrigation amount of 300 mm, 425 mm, 550 mm, 675 mm and 800 mm, respectively. ‘ F1 ‘, ‘ F2 ‘, ‘ F3 ‘, ‘ F4 ‘ and ‘ F5 ‘ indicate the fertilization amount of 280 kg·ha^-1^, 360 kg·ha^-1^, 440 kg·ha^-1^, 520 kg·ha^-1^ and 600 kg·ha^-1^, respectively. Different letters in the same column indicate the significant difference between treatments at P = 0.05 level. * and ** indicate significant difference at P = 0.05 and P = 0.01 levels, respectively. ns indicates non-significant difference at P = 0.05 level.

The amount of irrigation and fertilization greatly influences the fruit quality parameters. It can be seen from **Figure 4**, with the increase of fertilization amount and irrigation amount, SI, SW, SS and VC showed a trend of increasing first and then decreasing. Under the single effect of irrigation or fertilization amount. the SS reached the maximum under W4 irrigation and F3 fertilization, 16.18 and 16.07, respectively. Under the interaction of water and fertilizer, the maximum value of 16.80 was reached in W4F4 treatment, which was 3.83% and 4.54% higher than that of single factor. This shows that the interaction of water and fertilizer promotes the increase of SS in fruit and promotes the accumulation of sugar. Among other quality indexes, SW, SI and VC reached the maximum value under W4F4 treatment, which further indicated that the interaction of water and fertilizer played an important role in improving fruit quality. On the other hand, P and AT showed a trend of decreasing first and then increasing. Under the single factor of water and fertilizer, the P reached the minimum value of 10.27 kg·cm^-2^ and 10.54 kg·cm^-2^ under W5 irrigation and F3 fertilization. Under the interaction of water and fertilizer, the P of W5F5 treatment was the lowest, which was 10.01 kg·cm^-2^, Compared with the single factor of water and fertilizer, it was reduced by 2.60% and 5.29%, respectively. This shows that the interaction of water and fertilizer promotes fruit growth and development, resulting in a decrease in P at the same time. There were some differences in fruit quality among different treatments. The fruit SI was the largest under W4F3 and W5F4 treatments, and the fruit appearance quality was better. Under W4F4 treatment, the fruit SS was the highest, the fruit AT was the lowest, and the fruit taste was better. This shows that the sensitivity of fruit appearance quality and fruit taste to water and fertilizer is different. According to the analysis of variance, the effect of irrigation amount on apple quality index reached a very significant level (P<0.01). The effect of fertilization on SI, AT, VC, SW and SS reached a very significant level (P<0.01), and the effect on P reached a significant level (P<0.05). The synergistic effect of water and fertilizer had a significant effect on SS and AT (P<0.05), but had no significant effect on other quality indexes (**Figure 4**).

### 3.3 Yield, IWUE and PFP

**Figure 5:**
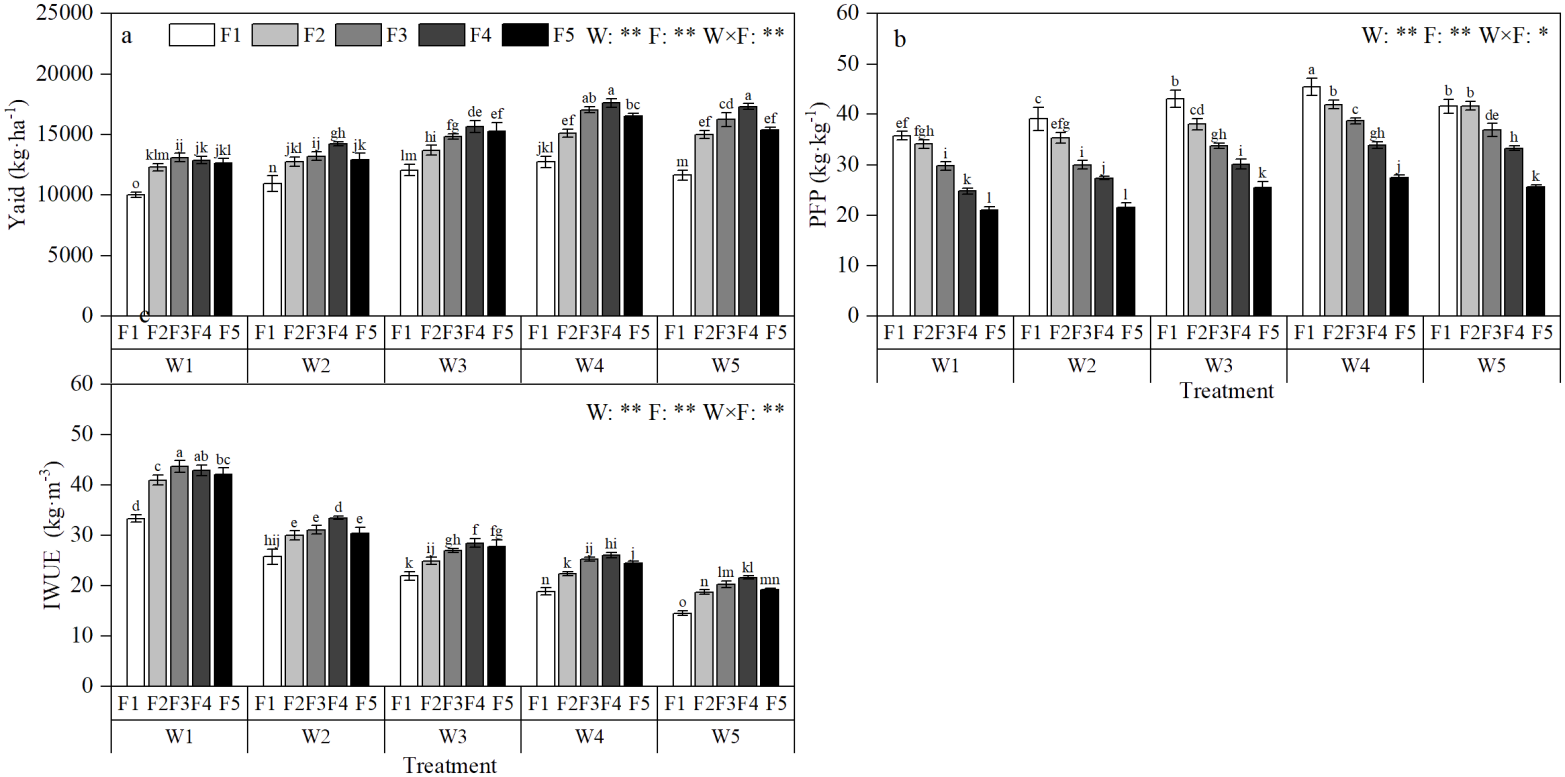
Effects of different irrigation amount and fertilization amount on the yield, irrigation water use efficiency, Productivity of fertilizer under magnetic-electric irrigation. Yield **(A)**, Irrigation water use efficiency **(B)**, Productivity of fertilizer **(C)**;’ W ‘ and ‘ F ‘ indicate the amount of irrigation and fertilization respectively. ‘ W1 ‘, ‘ W2 ‘, ‘ W3 ‘, ‘ W4 ‘ and ‘ W5 ‘ indicate the irrigation amount of 300 mm, 425 mm, 550 mm, 675 mm and 800 mm, respectively. ‘ F1 ‘, ‘ F2 ‘, ‘ F3 ‘, ‘ F4 ‘ and ‘ F5 ‘ indicate the fertilization amount of 280 kg·ha^-1^, 360 kg·ha^-1^, 440 kg·ha^-1^, 520 kg·ha^-1^ and 600 kg·ha^-1^, respectively. Different letters in the same column indicate the significant difference between treatments at P = 0.05 level. * and ** indicate significant difference at P = 0.05 and P = 0.01 levels, respectively. ns indicates non-significant difference at P = 0.05 level.

The amount of irrigation and fertilization greatly influences the yield, IWUE and PFP. It can be seen from **Figure 5A**, with the increase of irrigation and fertilization, the yield of apple increased first and then decreased. The yield of apple under W1 irrigation and F1 fertilization was lower, reaching 12584.42 kg·ha^-1^ and 13386.71 kg·ha^-1^, respectively. The yield of W4 irrigation and F4 fertilization was the highest, reaching 15957.81 kg·ha^-1^ and 15300.68 kg·ha^-1^, respectively. There under interaction of water and fertilizer, the yield of W4F4 treatment reached 17111.64 kg·ha^-1^, which was the highest yield treatment. Compared with single factor treatments, it increased by 7.23% and 11.83% respectively. There was no significant difference between W4F4 treatment and W4F3 and W5F4 treatments. In addition, there was a significant difference in apple yield compared with other water and fertilizer treatments (P< 0.05).Through the analysis of variance, it can be known that the effects of irrigation amount, fertilization amount and synergism of irrigation and fertilizer on yield reached a very significant level (P< 0.01) (**Figure 5A**). It can be seen from **Figure 5B**, with the increase of irrigation amount, IWUE showed a decreasing trend. Under the same fertilization amount, it showed a trend of increasing first and then decreasing with the increase of irrigation amount, and achieved the maximum value in W1F3 treatment. Except for W1F4, it showed significant difference with other treatments (P<0.05). According to the analysis of variance, the effects of irrigation amount, fertilization amount and synergism of irrigation and fertilizer on IWUE achieved a very significant level (P<0.01) (**Figure 5B**). It can be seen from **Figure 5C**, with the increase of fertilization amount, PFP decreased gradually. Under the same fertilization amount, PFP increased first and then decreased with the increase of irrigation amount. W4F1 treatment had the highest PFP, which was significantly higher than other water and fertilizer treatments (P<0.05). According to the analysis of variance, the effects of irrigation and fertilization on PFP achieved a very significant level (P<0.01), and the effect of irrigation and fertilizer interaction on PFP achieved a significant level (P<0.05) (**Figure 5C**).

### 3.4 Regression analysis

In the following analyses, the input irrigation amount and application amount were the independent variables; the upper and lower limits of irrigation inputs were those corresponding to the W1 and W5 levels, respectively; and the upper and lower limits of the application were F1 and F5 levels, respectively. Yield, IWUE, and PFP were selected as the response variables. Based on the least squares method, the binary quadratic regression equation was established using the matlab software. The correlation coefficients of the regression equation were greater than 0.85, and were greater than the critical correlation coefficient corresponding to the significance level of 0.05(R_0.05_ = 0.514), which indicated that it was reasonable to use the formula in the table to fit the apple tree yield, IWUE and PFP (**Table 3**). We calculated the amount of irrigation and fertilization required to maximize the above parameters through the regression model. The results showed that it was difficult to obtain the maximum yield, maximum IWUE and maximum PFP at the same time (**Table 4**).

**Table 3:** Regression equation of yield, IWUE, PFP for apple trees.

Dependent variable	Regression equation	*R^2^*	*P*
Yield	$Y_{1}=-0.02x_{1}^{2}-0.04x_{2}^{2}+24.49x_{1}+34.05x_{2}+0.01x_{1}x_{2}-1168.86$	0.86	0.01
IWUE	$Y_{2}=4.9\times 10^{-5}x_{1}^{2}-7.8\times 10^{-5}x_{2}-0.1x_{1}+0.07x_{2}+5.6\times 10^{-6}x_{1}x_{2}+49.7$	0.96	0.02
PFP	$Y_{3}=5.2\times 10^{-5}x_{1}^{2}+8.2\times 10^{-5}x_{2}^{2}+0.07x_{1}-0.14x_{2}-3.4\times 10^{-6}x_{1}x_{2}+58.6$	0.95	0.01

x_1_, irrigation amount (mm); x_2_, application amount (kg·hm^-2^); Y_1_, Yield (kg·hm^-2^); Y_2_, IWUE (kg·m^-3^); Y_3_, PFP (kg·kg^-1^).

**Table 4:** The maximum value of the relevant dependent variables and the amount of irrigation and fertilization.

Dependent variable	Maximum value	Corresponding irrigation amount (mm)	Corresponding fertilization amount (kg·ha^-1^)
Yield (kg·ha^-1^)	16219.21	699.98	519.19
IWUE (kg·m^-3^)	40.58	300.00	461.01
PFP (kg·kg^-1^)	49.58	678.79	280.00

**Figure 6:**
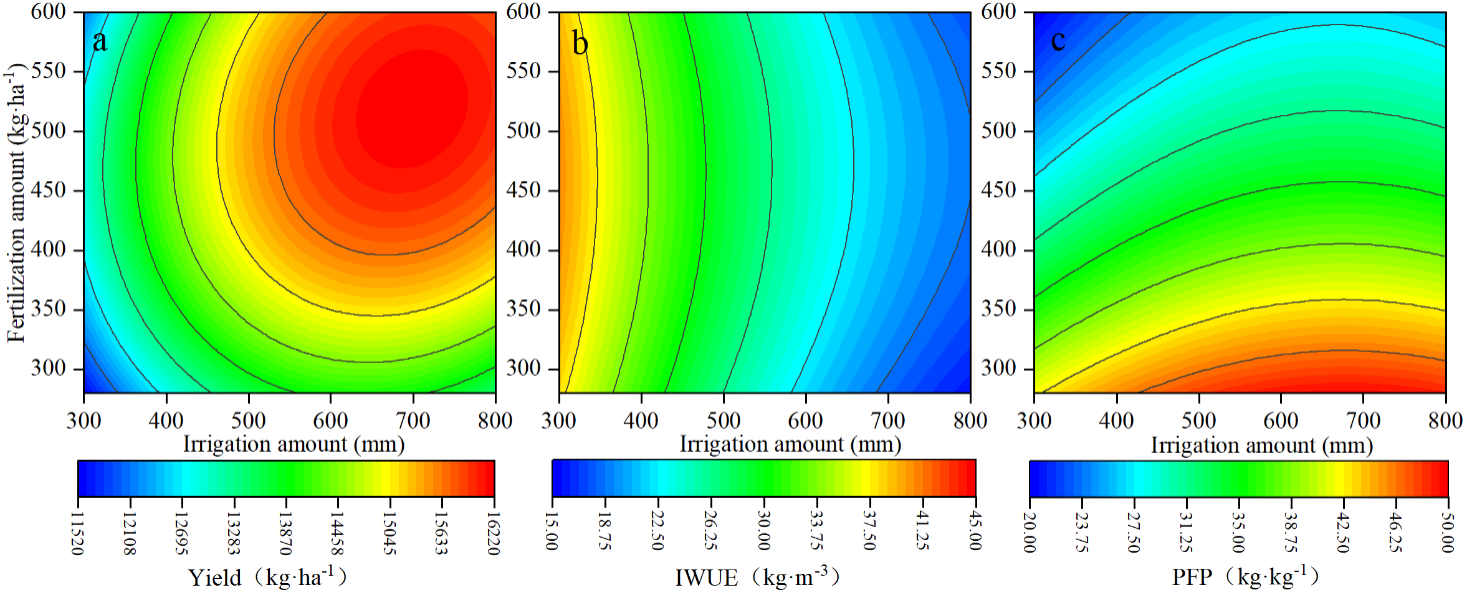
Regression fit of irrigation amount and fertilization amount inputs and yield, irrigation water use efficiency (IWUE) and partial factor productivity (PFP). **(A)**, the yield under different irrigation amounts and fertilization amount. **(B)**, the IWUE under different irrigation amounts and fertilization amount. **(C)**, the PFP under different irrigation amounts and fertilization amount.

Therefore, to help growers making the most suitable irrigation and application management strategies. We conducted a spatial analysis of apple tree yield, IWUE and PFP to determine which treatments had the best effect on water and fertilizer use and yield. Using the spatial analysis method. When the range of irrigation amount and fertilization amount was 418.21mm from 431.20 mm and 315.56 kg·ha^-1^ from 393.13 kg·ha^-1^, respectively, 90% of the maximum yield and 80% of the maximum IWUE and PFP could be achieved simultaneously (**Figure 6**).

### 3.5 Correlation analysis and principal component analysis

#### 3.5.1 Correlation analysis

**Figure 7:**
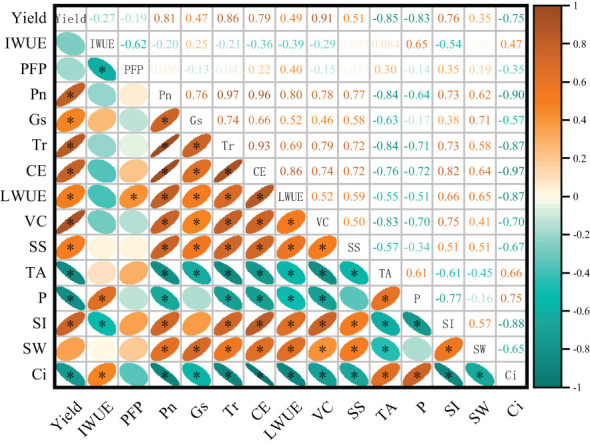
Pearson’s correlations among yield, quality, water and fertilizer use efficiency and other physiology parameters. The data was Pearson correlation coefficients (n = 25). Yield, apple yield; IWUE, irrigation water use efficiency; PFP, productivity of fertilizer; Pn, net photosynthetic rate; Gs, stomatal conductance; Tr, transpiration rate; CE, carboxylation efficiency; LWUE, leaf instantaneous water use efficiency; VC, vitamin C; SS, soluble solid; TA, titratable acid; P, Firmness; SI, shape index; SW, Fruit weight; Ci, intercellular CO_2_ concentratin; * and ** indicate significant difference at P = 0.05 and P = 0.01 levels, respectively. ns indicates non-significant difference at P = 0.05 level.

There were significant correlations between the yield, photosynthetic parameters and quality following treatments with magnetoelectric water and water and fertilizer coupling. There was a significant positive correlation between Pn, Ci, Gs, Tr, CE, LWUE and yield (P< 0.05). The absolute value of the correlation coefficient between yield and Pn exceeded 0.8. There was a negative correlation between TA and P and yield, and the absolute value of the correlation coefficient reached more than 0.8. There was a positive correlation between photosynthetic indexes such as Pn and SS, VC, SW, SI (P< 0.05), and a negative correlation with TA (P< 0.05). Therefore, apple leaf photosynthesis may be an important factor affecting apple yield formation and quality improvement. (**Figure 7**).

#### 3.5.2 Principal component analysis

It is well known that different combinations of irrigation and fertilizer and the magnetization and de-electronic treatment of irrigation water will affect the physiological state, yield, quality and water and fertilizer utilization efficiency of apples. If these effects are evaluated only by a single indicator, the results usually have certain limitations. If all the indicators are analyzed and evaluated, the number of variables is large and there are many correlations between variables, the evaluation results are quite different, and the complexity of the results analysis is increased. It is still difficult to determine the best combination of water and fertilizer. The principal component analysis method can concentrate the variables on the basis of not abandoning the complex information in multiple variables, and finally integrate them into a comprehensive index with fewer variables. Through the linear combination of multiple original variables, the principal component model is constructed to clarify the optimal treatment in the evaluation system. In this study, based on the principal component analysis method, the indicators related to yield, quality and water and fertilizer use efficiency were selected to optimize the 25 irrigation and fertilizer treatments under magnetized and de-electronation water irrigation, and the optimal irrigation amount and fertilization amount under magnetized and de-electronic water irrigation were determined.

It is necessary to test the suitability of sample data before using principal component analysis. The results show that the KMO value of the sample data is 0.646, which meets the requirement of greater than 0.5, indicating that the sample size is sufficient. The observed value of Bartlett sphericity test is 105, the accompanying probability P is less than 0.01, and the hypothesis of sphericity test is rejected, indicating that there is a correlation between the indicators, which is consistent with the results of correlation analysis. The sample data is suitable for principal component analysis (**Table 5**).

**Table 5:** KMO and Bartlett test.

KMO value	Bartlett test		
	Approximate chi-square	Degree of freedom	Significance
0.663	193.21	36	0.001

**Figure 8:**
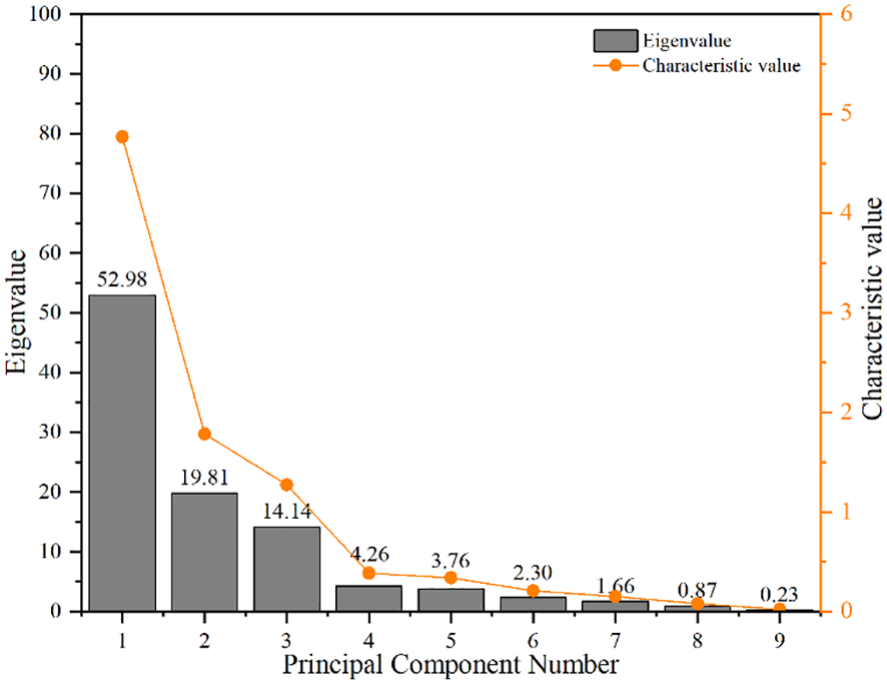
The scree plot of the principal component eigenvalues.

The results of principal component analysis showed that the eigenvalues of PC1, PC2 and PC3 were 4.768, 1.783 and 1.272, respectively, which all met the requirements of greater than 1, and the contribution rates reached 52.98, 19.81 and 14.14, respectively. The cumulative contribution rate was 86.93%, which met the requirements of greater than 75%. Therefore, the first three principal components can be selected to fully explain the coupling effect of magnetization and de-electronic water irrigation and different water and fertilizer treatments (**Figure 8**).

**Figure 9:**
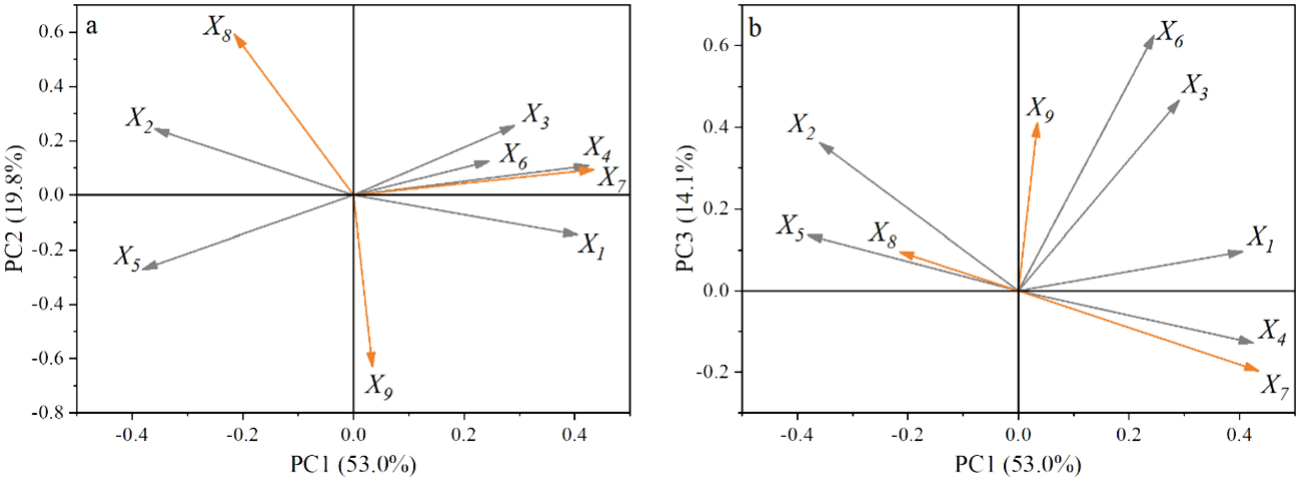
The principal component load diagram. PC_1_ is the first principal component, PC_2_ is the second principal component, PC_3_ is the third principal component; *X_1_* is the shape index, *X_2_* is the fruit firmness, *X_3_* is the soluble solid, *X_4_* is the vitamin C, *X_5_* is the titratable acid, *X_6_* is the fruit weight, *X_7_* is the apple yield, *X_8_* is the irrigation water use efficiency, *X_9_* is the productivity of fertilizer.

It can be seen from **Figure 9**, PC_1_ mainly included yield, SI, VC, AT and P. Among them, yield, SI and VC were mainly located in the positive half axis of PC_1_, and AT and P were mainly located in the negative half axis of PC_1_. The cumulative variance contribution rate of the five indexes reached 53.0%. PC_2_ mainly includes IWUE and PFP, in which IWUE is located in the positive half axis of PC_2_ and PFP is close to the negative half axis of PC_2_, and the cumulative variance contribution rate of the two reaches 19.8%. PC_3_ mainly includes SW and SS, both of which are close to the positive half axis of PC_3_, and the cumulative variance contribution rate reaches 14.1%. The magnetoelectric treatment combined water and fertilizer mainly affected apple yield, water and fertilizer use efficiency and vitamin C content related to quality (**Figure 9**).

Based on the results of principal component analysis, the evaluation model of water and fertilizer coupling effect of apple under magnetization and point water irrigation was constructed, as shown in Equations 5–8:


(5) $$PC_{1}=0.405X_{1}-0.360X_{2}+0.425X_{3}+0.291_{4}-0.381X_{5}+0.435X_{6}-0.215X_{7}+0.034X_{8}+0.245X_{9}$$

(6) $$PC_{2}=-0.145X_{1}\_0.243X_{2}+0.109X_{3}+0.255_{4}-0.271X_{5}+0.094X_{6}+0.591X_{7}-0.628X_{8}+0.124X_{9}$$

(7) $$PC_{3}=-0.172X_{1}+0.288X_{2}+0.129X_{3}+0.302_{4}-0.321X_{5}+0.111X_{6}+0.700X_{7}-0.744X_{8}+0.147X_{9}$$

(8) $$PC=0.610PC_{1}+0.228PC_{2}+0.162PC_{3}$$

Where, PC1, PC2 and PC3 represent the scores of each index under the first, second and third principal components respectively. X1 ~ X9 represent SI, P, SS, VC, AT, SW, Yield, IWUE and PFP, respectively. PC represents the comprehensive score under different treatments.

**Figure 10:**
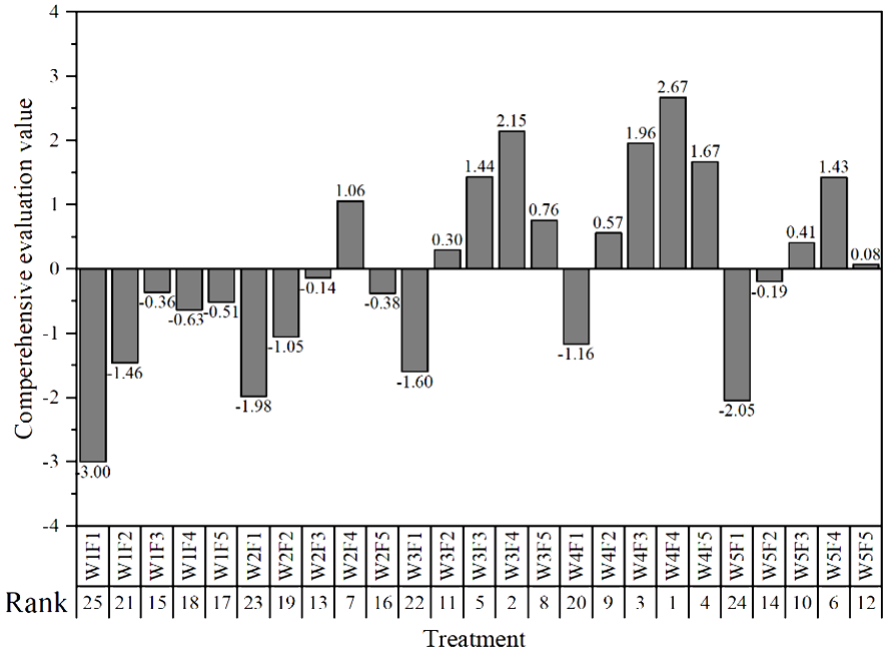
Comprehensive evaluation value. The first column label of the horizontal axis represents the experimental treatment, and the second column label represents the ranking of the comprehensive evaluation value.

The highest score treatment was the best water and fertilizer treatment under the experimental conditions. The comprehensive evaluation scores of W4F4, W3F4 and W4F3 reached 2.67, 2.15 and 1.96 respectively, which were the first three treatments with the highest comprehensive evaluation scores. The irrigation amount and fertilization amount were 675mm, 520 kg·ha^-1^,550mm, 520 kg·ha^-1^, 675mm and 440 kg·ha^-1^ respectively, indicating that the reasonable irrigation amount range under magnetic-electric water irrigation in Xinjiang was 550mm-675mm, and the fertilization amount range was 440 kg·ha^-1^-520 kg·ha^-1^ (**Figure 10**).

## 4 Discussion

### 4.1 Effects of different irrigation and fertilization on the photosynthetic characteristics

Photosynthesis is an important factor affecting crop productivity and provides the required nutrients for crop growth and development. Water and fertilizer are important factors affecting crop photosynthesis (Abdelaal et al., 2018; Hessini et al., 2019). Under magnetoelectric water irrigation, increasing the amount of irrigation and fertilization can improve the photosynthesis of plants. This promoting effect will gradually weaken with the increase of irrigation amount and fertilization amount. This may be because when the irrigation amount is low, the apple tree is affected by water deficit, the root water absorption capacity is reduced, the chloroplasts of the leaves are swollen, the arrangement is disordered, the thylakoid lamella is swollen or disintegrated, and the ultrastructure of the photosynthetic organs is destroyed. At this time, in order to reduce the water loss of the tree, the Gs of the leaves decreases, and the parameters such as Pn and Tr also show a low level (Chaves et al., 2009; Farooq et al., 2009). With the increase of irrigation amount, the activities of Ru BP carboxylase and photosynthetic carbon cycle enzyme in apple trees increased, the CO_2_ fixation ability of apple trees increased, and the photosynthesis also improved (Carmo-Silva et al., 2010; Kaiser et al., 2015). In addition, fertilization is also the main factor affecting the photosynthesis of apple trees. The study found that the lack of nitrogen in the tree will weaken the level of chlorophyll soluble protein and the synthesis and activity of photosynthetic enzymes, and reduce the photosynthetic capacity of the leaves (Sardans and Peñuelas, 2015). Similar to the results of this study, this study found that with the lowest fertilization treatment, with the increase of irrigation amount, the net photosynthetic rate of apple trees showed a trend of increasing first and then decreasing, but it was significantly lower than other fertilization treatments. When the amount of irrigation and fertilization was low, the plant growth was poor and the intercellular carbon dioxide concentration of plant leaves was high. The reason may be that water and nutrient deficiency and stress led to stomatal closure of leaves and decreased root water absorption capacity, decreased photosynthetic capacity of mesophyll cells, insufficient supply of adenosine triphosphate (ATP) and reduced nicotinamide adenine dinucleotide phosphate (NADPH), increased Ci, and thus photosynthesis was affected (Kalaji et al., 2014). The change of Ci can be used as the basis for judging whether the decrease of photosynthetic rate is dominated by stomatal factors or non-stomatal factors. The results showed that when the soil moisture content decreased, the water absorption rate of roots decreased, resulting in the decrease of water in fruit trees and the active decrease of Gs in leaves to adapt to the changes of surrounding environment. The significant decrease of Tr increased LWUE, which was different from the results of this study. This study found that LWUE increased first and then decreased with the change of irrigation amount, which may be caused by the decrease of photosynthesis-related enzyme activity and the weakening of photosynthesis caused by water deficit (Zivcak et al., 2013).

### 4.2 Effects of different irrigation and fertilization on the fruit quality

Fruit quality is an important index to determine the nutritional value and economic benefits. With the improvement of people’s living quality, agricultural production pursues high yield and high quality. The contents of vitamin C, soluble solids, titratable acid, firmness, fruit shape index are the most important indexes to evaluate the quality of apple (Zha et al., 2023). Fertilization and irrigation is an important way to improve quality, and different quality indicators have different responses to irrigation and fertilization (Han et al., 2023). Under low irrigation, SS and VC are significantly lower than other water and fertilizer treatments, which is consistent with previous research results (Chen et al., 2014; Zhang et al., 2023b). Less irrigation amount could reduce the transportation of phloem juice to the fruit, resulting in a decrease in the water content of the fruit, and hence the solute concentration is relatively increased (Tao et al., 2023)。In the current study, The fruit hardness increased significantly under low irrigation treatment, and was significantly higher than other water and fertilizer treatments, which was opposite to the quality indexes such as SS and VC. It is generally believed that the change of fruit hardness can reflect the degree of maturity of the fruit. Studies have shown that when the fruit gradually matures, the fruit hardness will gradually decrease (Maatallah et al., 2024). Under low irrigation, in order to maintain the normal physiological state, the tree distributes most of the water to vegetative growth, and the water supply to maintain the growth and development of the fruit is insufficient, and the fruit development is slow, resulting in an increase in fruit hardness (Musacchi and Serra, 2018). In this case, it is more conducive to the transportation and storage of fruits. In our communication with managers, we found that after the fruit trees entered the mature stage, people often reduced the amount of irrigation or did not irrigate to maintain the hardness of the fruit. Similar conclusions have been drawn in previous studies (Wang et al., 2019b). Appropriate fertilization application rate can improve fruit quality, but excessive fertilization amount will cause quality decline. The contents of VC, SS, SW and SI significantly increased with the increase of fertilization amount in our study. This may be because, in the case of increasing the amount of fertilizer, the supply of nutrients necessary for plant growth and development such as nitrogen, phosphorus and potassium increased, which increased the photosynthetic enzyme activity and protein synthesis of plants (Cheng et al., 2021). However, when the amount of fertilizer increased to a certain extent, the contents of SS, VC, SI and SW reduced, yet the content of TA increased. Excessive fertilization affected the absorption of nutrients by fruit trees, but increased the synthesis of titratable acid-related proteases (Zhang et al., 2023a). Similar research results support this study (Zhou et al., 2019; Zhang et al., 2023b). Additionally, SI is an important index of fruit appearance quality, SI reached the maximum value in W4F3。Studies have shown that fruits often have good appearance quality under appropriate water and fertilizer supply levels (Yang et al., 2023). This also shows that the quality of apple fruit is acceptable when the irrigation amount and fertilization amount are 675 mm and 440 kg·hm^-2^ respectively.

### 4.3 Effects of different irrigation and fertilization on the yield, IWUE and PFP

In agricultural production, reasonable irrigation and fertilization can coordinate the distribution of crop nutrients, promote the synthesis of photosynthetic products, and improve crop yield and water and fertilizer use efficiency (Mueller et al., 2012). The current research shows that reasonable irrigation amount and fertilization amount have obvious yield-increasing effect, but the yield-increasing effect gradually weakens with the increase of irrigation amount and fertilization amount. This may be because the appropriate irrigation amount has a positive effect on creating a root soil environment, improving the root activity of apples, strengthening the water absorption capacity of roots, and satisfying the water demand of trees (Rolli et al., 2015). In addition, water is the main carrier of tree nutrient transport. Under the appropriate water supply, the transport and distribution of tree nutrients are more reasonable, thereby increasing apple yield (Stape et al., 2010). Researchers have achieved similar results in the study of related fruit trees such as grapes and pomegranates (Parvizi et al., 2014; Ma et al., 2023).

Less fertilizer application may directly lead to insufficient supply of fruit nutrients, thereby reducing yield. While the excessive amount of fertilization leads to a large amount of nutrients being used for the growth of fruit trees, and the nutrient distribution is unreasonable, which inhibits the development of fruit and affects the yield (Ning et al., 2023). The study found that reasonable fertilization increased the soil respiration rate of mango at different growth stages and improved the absorption and transport of nutrients by roots (Sun et al., 2022). Compared with other fertilization treatments, the growth and leaf area index of apple saplings increased to varying degrees under higher fertilization treatments, but the yield of apple did not reach the maximum (He et al., 2023). Therefore, the appropriate amount of fertilizer can not only meet the nutrients required for the growth of fruit trees, but also maintain the ideal photosynthetic capacity, so as to increase the yield of fruit trees. At the same time, the lack of nutrient supply may lead to the weakening of tree resistance and the increase of pests and diseases, which will affect the yield and water and fertilizer utilization efficiency.

The synergistic effect of water and fertilizer increased yield and water and fertilizer use efficiency. Under low water and fertilizer treatment, with the increase of irrigation amount and fertilization amount, the difference between treatments was small. Under high irrigation amount and fertilization amount, the coupling effect between water and fertilizer was strengthened, which could better reflect the mutual promotion between the two. Reducing irrigation amount and fertilization amount was beneficial to promote the improvement of water and fertilizer use efficiency (Parvizi et al., 2014). When the irrigation amount was reduced and the fertilization amount was increased, the IWUE was further improved, increasing the irrigation amount and reducing the fertilization amount, the PFP increased significantly. It show that PFP was positively correlated with irrigation amount and negatively correlated with fertilization amount. IWUE was negatively correlated with irrigation amount and positively correlated with fertilization amount. It is further explained that there is a close relationship between water and fertilizer, and the rational use of the relationship between water and fertilizer and the optimization of water and fertilizer management play an important role in improving the utilization efficiency of water and fertilizer (Dai et al., 2021). By establishing a regression model, it was found that when the apple yield, IWUE and PFP reached the maximum value respectively, corresponding to different irrigation and fertilization amounts, it was impossible to take into account the goal of the highest yield and the maximum utilization efficiency of water and fertilizer at the same time. When the yield regression model obtained the optimal solution, the irrigation amount and fertilization amount were 699.98 mm and 519.19 kg·ha^-1^, respectively. When the IWUE and PFP regression models obtained the optimal solution, the irrigation amount and fertilization amount were 300 mm, 461.01 kg·ha^-1^ and 678.79 mm, 280.00 kg·ha^-1^, respectively, which were quite different from the optimal solution of the yield regression model. These results indicate that the increase of yield is often inversely proportional to the increase of IWUE and PFP. Under the amount of irrigation and fertilization, the distribution of dry matter changes, the proportion of nutrient distribution decreases, and the proportion of photosynthetic assimilates to fruits increases (Wu et al., 2023).

## 5 Conclusion

Under the same irrigation amount, with the increase of fertilization amount, Ci decreased first and then increased. With the increase of irrigation and fertilization, Pn, Tr, Gs, CE and LWUE increased first and then decreased. Apple quality was significantly affected by irrigation and fertilization (P<0.05). The synergistic effect of water and fertilizer on soluble solids and titratable acid reached a significant level (P<0.05). There is an inverse relationship between the yield and IWUE and PFP. When the yield reaches the maximum, IWUE and PFP are lower. There was a negative effect between IWUE and irrigation amount, and there was also a negative effect between PFP and fertilization amount.

Correlation analysis showed that there was a certain correlation between photosynthetic characteristics and yield and quality. The results of regression analysis showed that the change trend of yield with irrigation and fertilization was in line with the binary quadratic regression model, and the maximum value could reach 16219.21 kg·ha^-1^. When the yield reached the maximum value, the irrigation and fertilization were 699.98 mm and 519.19 kg·ha^-1^, respectively.

The results of principal component analysis showed that the comprehensive evaluation scores of W4F4, W3F4 and W4F3 reached 2.67, 2.15 and 1.96, respectively, which were the first three treatments with the highest comprehensive evaluation scores. The reasonable irrigation amount under magnetic and de-electric water irrigation was between 550 mm and 675 mm, and the fertilization amount was between 440 kg·ha^-1^ and 520 kg·ha^-1^.

This study provides valuable guidance for improving water and fertilizer productivity, crop yield and quality in arid areas of Xinjiang by using magnetized and de-electrolyzed water irrigation.

## Data availability statement

The original contributions presented in the study are included in the article/supplementary material. Further inquiries can be directed to the corresponding author.

## Author contributions

XD: Writing – original draft, Writing – review & editing. QW: Conceptualization, Funding acquisition, Writing – review & editing. WM: Investigation, Software, Writing – review & editing. XW: Resources, Writing – review & editing.

## Glossary

AFS: Anthesis fruit setting stage
YFS: Young fruit development stage
FES: Fruit expansion stage
FRS: Fruit ripeness stage
Pn: Net photosynthetic rate
Gs: Stomatal conductance
Tr: Transpiration rate
Ci: Intercellular CO_2_ concentratin
LWUE: Leaf instantaneous water use efficiency
CE: Carboxylation efficiency
IWUE: Irrigation water use efficiency
PFP: Partial factor productivity
VC: Vitamin C
SS: Total soluble solids
TA: Titrated acidity
P: Firmness
SI: Fruit shape index
SW: Single fruit weight
